# The special algorithm based on RSA cryptography for signing and verifying digital signature

**DOI:** 10.1016/j.heliyon.2025.e42481

**Published:** 2025-02-07

**Authors:** Kritsanapong Somsuk

**Affiliations:** Department of Computer and Communication Engineering, Faculty of Technology, Udon Thani Rajabhat University, UDRU, Udon Thani, Thailand

**Keywords:** RSA, Digital signature, Modular multiplication, Modular square, Signing process, Verifying process

## Abstract

This aim of this research is to present the improved equations to enhance RSA cryptography for signing and verifying processes. The primary benefit is in signing procedure, where it could decrease computing resources by introducing a new integer to be utilized as exponents instead of the private key. In fact, this integer is always smaller than the private key. However, authenticating the digital signature will require additional processing resources. The result obtained from validating the signature with the original equation must be multiplied by a single integer. However, while evaluating the entire process of signing and validating digital signatures, the proposed method presents a chance to significantly reduce the computing resources. Furthermore, two new techniques for generating all necessary parameters are introduced, Algorithm 3.1 and Algorithm 3.2. Although the long duration of these procedures is required, they will be executed only once. The experimental results demonstrate that Algorithm 3.2, which has a longer execution time than Algorithm 3.1, demands an average period of 70 s to generate a 1024-bit modulus and all associated parameters. Furthermore, the experimental results on the process of signing digital signatures indicate that the proposed method may reduce the average time about 30 %.

## 1 Introduction

Cryptography [1] involves the methodologies employed to protect confidential information by utilizing encryption and decryption procedures. The internet is extensively used as a communication medium nowadays because of its simplicity and rapidity [2]. However, it provides security risks by allowing intruders to intercept data sent through the network [3]. Cryptography is employed to improve the security of sensitive information by encrypting the original data to get the ciphertext which is an unreadable message. The ciphertext is transmitted to the recipient instead of sending the original plaintext. After receiving the ciphertext, the recipient decrypts it to get the plaintext. In fact, if attackers intercept data transferred across the network, they would only read the ciphertext. However, this value is useless unless they can compute the secret key or the private key. In digital computing, cryptography is classified into two main categories: The primary category is symmetric key cryptography [4] utilizing the secret key for both encryption and decryption processes. The benefits of this system are strong security protocols and fast processing capabilities. On the other hand, a major challenge is the secure transmission of the secret key between the sender and receiver, especially when they are extremely far away. AES [5,6] and Triple DES [7,8] are two frequently utilized symmetric key algorithms identified for their reliability and effectiveness. Asymmetric key cryptography [9], typically referred to public key cryptography, is a type of cryptography that differs from symmetric key cryptography by using two separate keys for the processes of encryption and decryption. The public key is available to everyone in group. The receiver keeps the private key to decrypt the encrypted message. Asymmetric key cryptography has significant benefits, including improved security and the capability to resolve the key exchange problem found in symmetric key systems.

Digital signature generation and verification [10,11] are a method used to verify the identification of the individual. In addition, some public key cryptography techniques may be used for this task. The key generator utilizes the private key to digitally authenticate the electronic document. Afterwards, both the digitally signed message and the actual document are sent to be verified. The verifier applies the public key to validate the digital signature of the message, then comparing the result with the original message. If the values are the same, the validity is confirmed. On the other hand, if the results are inconsistent, this data set will be eliminated. RSA [12] is a robust method in asymmetric key cryptography. Indeed, strong prime numbers must be chosen to generate the modulus to guarantee the security level. In fact, the modulus length must be a minimum of 1024 bits [13]. In addition, this technique may be used for data security and digital signature generation [14]. However, the main obstacle is in the generation of the large private key to protect against adversaries [15], resulting in extended computing time for signing process. Therefore, several techniques have been proposed to reduce the computation time. One of these methods is the applying of Chinese Remainder Theorem (CRT) [16,17] with RSA by dividing the private key into smaller components. The next technique is Square and Multiple Algorithm [18], which involves converting the exponent into binary form and performing calculations using Modular Squaring and Modular Multiplication. The quantity of computational resources is based on the number of bits in the exponent.

The advancement of digital signature generation and verification is ongoing, primarily focused on improving security against potential risks, especially those presented by high-performance quantum computers. The enhancement of digital signature development and verification is seen in Ref. [19]. This study presented a quantum e-commerce system. It focuses on demonstrating the viability and robustness against flawed devices, while attaining a realistic signature rate and agreement size appropriate for safe e-commerce transactions. This framework emphasizes the capability of quantum cryptography in tackling security issues in e-commerce. In addition, if high-performance quantum computers are fully developed, Shor's Algorithm [20] is a quantum algorithm capable of breaking RSA. Hence, developing digital signature and verification systems resilient to harm from quantum computer algorithms is an important problem. Unconditionally Secure Quantum Digital Signatures (QDS) [21,22] represents an alternative technique for signing and verifying digital signatures securely through processing with quantum computers. QDS uses the principles of quantum mechanics to ensure information-theoretic security, setting it apart from traditional digital signatures that rely on computational assumptions. Relevant quantum properties, including unforgeability, non-repudiation, and transferability, provide QDS a compelling option for applications in the post-quantum age. However, the actual execution of QDS encounters several substantial obstacles, such as the complexity of managing quantum states, the need for long-term quantum memory, and the volatility of quantum communication channels. While advancements such as the use of coherent states and non-destructive state verification have mitigated some of these issues, current systems remain unsuitable for widespread deployment. Additionally, the lack of standards and security proofs against coherent attacks remains a critical area for further research. Although QDS holds long-term potential, these challenges prevent the technology from fully replacing traditional digital signature methods in the short term. These limitations underscore the necessity for improving digital signatures on digital computers to address security concerns.

The aim of this study is to present the modified method improved from RSA for signing and verifying processes. The small integer is chosen as the new key to replace the private key. It is always smaller than the private key. Then, it implies that the signing process requires less processing resources because of the smaller exponent. However, the multiplication of the outcome from the traditional equation and an integer generated by using modular exponentiation with a small exponent is necessary for the new verification equation. Then, the verification process requires a small increase in processing resources relative to the original equation. Nevertheless, if all parameters under conditions suitable for the proposed method are established, it is very possible that the proposed method demonstrates higher computational efficiency relative to RSA regarding overall resource use for both signing and verifying processes. Furthermore, the proposed method may be improved by applying with methods such as Chinese Remainder Theorem (CRT) and the Square and Multiply Algorithm to speed up the process.

## 2 Related works

This section will provide an overview of the literature relevant to the area of this research. The topic will include RSA scheme and techniques for accelerating the processes of digital signature generation and validation. In fact, this study is focused on improving the procedures for signing and validating digital signatures, followed by an analysis of RSA's concept for generating and verifying the digital signatures for electronic documents.

### 2.1 RSA

RSA is the public key cryptography introduced by Ron Rivest, Adi Shamir, and Leonard Adleman in 1977. Its strength lies in its applicability to both data security and digital signature generation. The process begins by generating the parameters required for the signing and verification processes, including five different steps. The procedure to create parameters is as follows:

Step 1 Randomly select two large prime numbers, denoted as *p* and *q.*
Step 2 Compute the modulus (*n*), *n* *=* *pq*
Step 3 Compute Euler's totient function (Φ *(n)*)*,* Φ *(n) = (p-1)(q-1)*
Step 4 Choose the public key (*e*) satisfying *1 < e <* Φ *(n)* and *gcd(e,* Φ *(n))* *=* *1*
Step 5 Compute the private key (*d*) from the following equation: *d* *=* *e*^*−1*^ *mod* Φ *(n)*

After generating all parameters, the key generator publicly discloses *e* and *n*, while maintaining the privacy of *p*, *q*, *d*, and Φ *(n)*. Assume that the individual approved as the key generator, possessing an electronic document (*m*), wishes to establish identity. Equation (1) can be selected to represent the encryption of *m*.

(1) $$s=m^{d}\;\mathrm{mod}\;n$$

where *s* represents as the encryption of *m*, which has been signed by key generator. After *s* is generated, *s* and *m* are transmitted to the verifier, who verifies the digital signature by using equation (2).

(2) $$m^{\prime }=s^{e}\;\mathrm{mod}\;n$$

The verifier then compares *m'* with *m*. If both values are identical, it confirms that *s* is really the digital signature produced by the actual owner of the document. On the other hand, the verifier will decline the data whenever *m'* does not match *m*.

Because of the various sizes of *m* in practical applications, a hash function [23] is frequently applied to generate hash values with the same size. Before the signing process, the sender computes the hash value (*h*) of *m* and then signs *h* instead of *m*. Let *s'* represent the encryption of *h*, the sender transmits the *s'* and *m* to the verifier, who uses equation (2) to verify *s'*. The verifier further calculates the hash *h* of *m*. Finally, the result from verifying process is compared to *h*. If both values are the same, *s'* is confirmed as the valid digital signature. The verifier will reject the data when the outcome of the verification procedure does not match *h*. However, the discussion on hash functions will be removed throughout the presentation of the proposed method. In fact, hash functions may often be used in practical situations.

The security of RSA relies on the difficulty of factoring *n*. If *n* is sufficiently large, it would be difficult to derive *p* and *q*, even with the most advanced computer systems today. The task will require an extensive amount of time. Currently, if *n* has a minimum of *1024* bits, no technique currently exists that can factor *n* in polynomial time.

### 2.2 Hamming weight

Hamming Weight [24] refers to the number of bits equal to *1* in the binary representation of an integer. For example, Hamming Weight of *17* is *2*, because *17* can be expressed as *10001*_*2*_. In fact, Hamming Weight of the exponent directly influences the quantity of Modular Multiplication operations required for Modular Exponentiation. An increment of Hamming Weight of the exponent requires further Modular Multiplication computations. On the other hand, a low Hamming Weight of the exponent can decrease the number of Modular Multiplication computations.

### 2.3 Square and multiple algorithm

Square and Multiple Algorithm is employed to effectively calculate Modular Exponentiation. The main computational resources include Modular Squaring and Modular Multiplication operations.

Let *c* *=* *a*^*b*^ *mod* *n*, before calculating *c* by using Square and Multiply Algorithm, the first step is to convert *b* into its binary representation. The binary of *b* will be considered to determine the number of Modular Squaring and Modular Multiplication. However, the number of Modular Squaring operations is determined by the count of bits in *b* when it is equal to *1*, whereas the number of Modular Multiplication operations depends on the Hamming Weight of *b*. Algorithm 2.1 illustrates the calculation of *c* *=* *a*^*b*^ *mod* *n* using Square and Multiple Algorithm.

Algorithm 2.1 Square and multiple algorithm.

**Figure:**
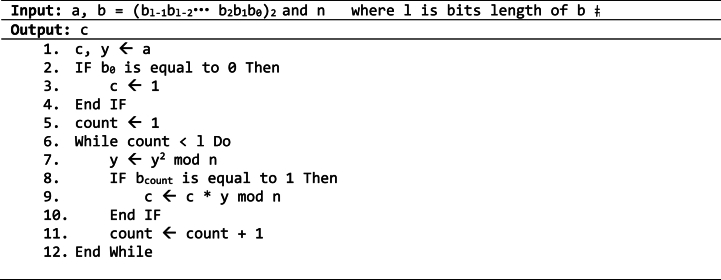

### 2.4 Chinese Remainder Theorem

Chinese Remainder Theorem (CRT) is a mathematical theory that can be employed for computing Modular Exponentiation by breaking down the large exponent into smaller components. Each component serves as an exponent for different Modular Exponentiation computations, with the various prime factors of *n* serving as the divisors of each process. Therefore, CRT might be employed only for the task of digitally signing signatures, because this function is performed by the key generator possessing all the private information. Let *m* represent an original message intended for the signing process. The process for executing CRT with RSA for digitally signing and verifying a signature is detailed below:

The procedure starts by splitting *d* into many smaller components, the number of components based on the number of prime factors of *n.* However, this research indicates that *n* has two prime components. Therefore, *d* is partitioned into two components, *d*_*p*_ and *d*_*q*_, by using equation (3) and equation (4).

(3) $$d_{p}=d\;mod\;p\;–\;1$$

(4) $$d_{q}=d\;mod\;q\;–\;1$$

The next step is to compute *s*_*p*_ and *s*_*q*_ using equation (5) and equation (6).

(5) $$s_{p}=m^{d_{p}}\;mod\;p$$

(6) $$s_{p}=m^{d_{q}}\;mod\;q$$

Let *y*_*p*_ = *p*^*−1*^ *mod* *q* and *y*_*q*_ = *q*^*−1*^ *mod* *p*, the final step is to compute *s* by using equation (7).

(7) $$s=\left(s_{p}y_{q}q+s_{q}y_{p}p\right)\;\mathrm{mod}\;n$$

### 2.5 Special algorithms to speed up RSA decryption process

This section analyzes methods utilized to reduce the processing time required for decryption process. The term of Special Algorithm indicates the employing of an alternative equation structure, it is different from equation (1). This alternate equation form produces the same result as equation (1). In fact, an alternative equation will be implemented when it is considered to the most effective computing resources, considering the number of Modular Square and Modular Multiplication operations. However, these solutions may be efficient only under conditions.

In 2017, the author found that equation (8) may be utilized for RSA decryption by using *x* as the exponent instead of *d* and calculating the Modular Inverse (*c*^*−1*^ *mod* *n*) [25]. The relationship between *x* and *d* is expressed by the equation *x* *=* Φ *(n) - d*, where *n* represents a numerical value. Therefore, if *d* is defined as a large integer, then *x* will be reduced, providing equation (8) suitable for decryption process.

(8) $$m={\left(c^{-1}\right)}^{x}\;\mathrm{mod}\;n$$

where *m* is the original message and *c* is the ciphertext.

In addition, equation (9) can be selected to sign the signature.

(9) $$s={\left(m^{-1}\right)}^{x}\;\mathrm{mod}\;n$$

In 2021, another equation for RSA decryption was proposed by expressing *d* as the difference between two integers, *d* *=* *a - b,* where *a, b* ∈N [26]. In fact, decrypting the encrypted message by using equation (10) is very efficient when the bit values with a value of *1* in *d* are arranged into substantial consecutive clusters.

(10) $$m=c^{a}c^{-b}\;\mathrm{mod}\;n$$

In 2023, a new integer (*d*_*r*_) [27] was proposed to speed up the process. In fact, the integer suitable for the condition must exceed *d* and has a reduced Hamming weight. This condition implies an increase in the number of Modular Square computations while significantly reducing the number of Modular Multiplication operations. Let *j* ∈N, then *d*_*r*_ can be obtained from *d*_*r*_ = *d* *+* *j* Φ *(n)* to be used as the exponent instead of *d*.

In fact, each approach in this section may be applied with CRT to speed up the signing process. Therefore, it is recommended to choose the most effective techniques for digital signatures [28]. When *n* is generated from two prime numbers, the procedure has two different elements: establishing the best approach for computing *m*_*p*_ and finding the most efficient technique for calculating *m*_*q*_. These values are then utilized to determine *m*.

### 2.6 Security of RSA

At present, RSA is one of the algorithms targeted by attackers. The security is based on the difficulty of integer factorization. Currently, many factorization methods have been developed. The efficiency of each method is different. For examples, Trial Division Algorithm (TDA) [29,30], which demonstrates high efficiency when at least one factor is relatively small or close to $\sqrt{n}$. Fermat Factorization Algorithm (FFA) [[31], [32], [33]] is suitable to be selected to factorize *n* when the factors are close to each other. Shor's Algorithm is an efficient quantum algorithm that identifies the period of a function as an initial phase for factorization on a classical computer. This implies that if high-performance quantum computers are fully developed, RSA security will be rendered obsolete. However, while quantum computers remain in the developmental phase, without a firm completion timetable (perhaps extending beyond 10 years), RSA continues to be widely recognized and employed. Moreover, other factorization algorithms have been proposed [34,35]. However, to prevent RSA from being attacked by using factorization, it is essential to use a large *n* that includes strong prime integers.

In addition, continued fractions offer another mathematical approach for attacking RSA. In 1990, M. Wiener [15] introduced a method known as Wiener's Attack, which applies continued fractions to compute *d*. If *d* is less than *n*^*0.25*^, then it is very possible to finish a task within polynomial time. Later, many researchers have proposed the improvements of Wiener's Attack to enhance its effectiveness. For example, in 1999, D. Boneh and G. Durfee [36] refined the method, demonstrating that *d* could be rapidly computed when it is smaller than *n*^*0.292*^. In 2002, B. de Weger [37] further optimized the equations for computing continued fractions, replacing the term $\frac{e}{n}$ with $\frac{e}{n-2\sqrt{n}+1}$. In this research, it shows that |p−q| must be assigned at least of the order of $\sqrt{n}$ and *d* must be larger than $n^{\frac{1}{3}}$ to maintain high levels of security. In 2020, the modified algorithm based on continued fraction was proposed [38]. This work expands on previous methodologies, such as Wiener's attack and de Weger's attack, generalizing existing techniques and exhibiting enhanced efficiency under certain situations. The research presents novel methodologies for parameter selection and approximation, using sequences and rational numbers to improve attack efficacy. Empirical examples demonstrate the method's efficacy in situations when conventional techniques are inadequate. This study enhances the comprehension of RSA weaknesses and emphasizes the need for stringent parameter selection in RSA. Therefore, to avoid computing *d* rapidly by using Wiener's attack or the modified algorithm, a large value of *d* should be selected.

## 3 The proposed method

The aim of this study is to present an improved algorithm modified from RSA for signing and verifying digital signatures. The main goal is to optimize computing resources in Modular Multiplication and Modular Square operations. In fact, the approach is accomplished by using the new equations for the generation and verification of digital signatures. The advancement involves the utilization of a new exponent for the signing process. This number has a smaller size in comparison to *d*, resulting in reduced processing resource requirements. In addition, the new exponent may be either an even or odd integer, in contrast to *d*, which is only an odd positive integer. However, the process of verifying digital signatures demands a little greater amount of computer resources.

The proposed method is different from the methods presented in Section 2.5, because the outcomes derived from the application in this section will use equation (2) for verifying process. However, the results derived from the generation of digital signatures using the proposed method will exclusively use the new provided equation for signature verification.

Theorem 1 Given that Φ *(n)* *=* *eb* *+* *x where b* and *x* are a positive integer, it can be inferred that the equation (11) can be utilized for signing *m*, and the equation (12) can be used to verify to confirm that *m* has been digitally signed by the actual signer.

(11) $$j=m^{b}\;\mathrm{mod}\;n$$

(12) $$m=j^{e}m^{x+1}\;\mathrm{mod}\;n$$

**Proof:**

This proof shows that the utilization of equation (11) and equation (12) for signing and verifying digital signatures will consistently provide *m*.

From, *j*^*e*^*m*^*x+1*^ *mod* *n = (m*^*b*^*)*^*e*^*m*^*x+1*^ *mod* *n*

*=* *m*^*eb*^ *m*^*x+1*^*mod* *n*

Because, Φ *(n)* *=* *eb* *+* *x*

Then, *eb* *=* Φ *(n) – x*

From Euler's Theorem, $m^{\Phi \left(n\right)}$ *mod* *n* *=* *1*

Then, *m*^*eb*^ *mod* *n* *=* $m^{\Phi \left(n\right)-x}$ *mod* *n*

$$=m^{\Phi \left(n\right)}\;m^{-x}\;\mathrm{mod}\;n$$

If *m*^*x+1*^ is multiplied both sides, then

$$m^{eb}m^{x+1}\;\mathrm{mod}\;n=m^{\Phi \;\left(n\right)}\;m^{-x}\;m^{x+1}\;\mathrm{mod}\;n$$

$$=m^{\Phi \;\left(n\right)}\;m\;\mathrm{mod}\;n$$

= *m*

Therefore, if *m* is signed by using equation (11), it can be recovered by using equation (12).

It is important to maintain *x* at a small number to prevent unnecessary time consumption during the verification procedure. In contrast to using the original equation for verifying process, the proposed method requires more resources, because it involves multiplying equation (2) by *m*^*x+1*^. On the other hand, when comparing *b* and *d*, it can be observed that *b* is smaller than *d* because of the equation: *ed* *=* *k* Φ *(n) + 1*, where *k* ≥ *1*, and *eb* *=* Φ *(n) - x*. For an equivalent value of *e*, *d* is always higher than *b*. Furthermore, if *k* is large, then *d* is substantially higher than *b*.

Example 1 Suppose the key generator selects *p* *=* *28183*, *q* *=* *30011*, then *n* *=* *845800013* and Φ *(n)* = *845741820*. If *e* *=* *13949* is chosen, then d *=* 845681189. Find *b* and *x* that may replace to *d*.

**Sol:** From, Φ *(n)* *=* *eb* *+* *x*

Then, *845741820* *=* *13949b* *+* *x*

If *b* *=* *60631* and *x* *=* *1* are selected, then

$$\left(13949\right)\left(60631\right)+1=845741820=\Phi \left(n\right)$$

Therefore, the equations for digital signature generation and verification are:

$$j=m^{60631}\;\mathrm{mod}\;845800013$$

$$m=j^{13949}\;m^{2}\;mod\;845800013$$

Suppose *m* = *17*, the encrypted message can be calculated as follows:

$$j=17^{60631}\;mod\;845800013=140055915$$

After sending *j* = *140055915* and *m* = *17* to the recipient, the recipient can verify the digital signature by using the following equation:

$$m=\left(140055915^{13949}\right)\;\left(17^{2}\right)\;mod\;845800013=17$$

Because of the significant difference in size between *b* and *d*, using equation (11) for signing process leads to decrease the processing resources. Regarding the minimal value of *x*, the verification of the signature by using equation (12) results in just a small rise in computing cost.

From *p* and *q* in example 1, assuming *e* and *d* are changed to *883* and *338105167*, respectively. It implies that *b* = *957805* and *x* = *5* can be chosen, because *(883)(957805) + 5* *=* *845741820* = Φ *(n)*. However, it is observed that *b* is smaller than *d*, leading to reduced computational cost. Furthermore, if *e* = *431* and *d* = *421889771* are established, then *b* = *1962278* and *x* = *2* may be chosen. Therefore, it may be inferred that *x* and *b* may be designated as even positive numbers.

The proposed method for signing and verifying processes involves three distinct procedures: key generation, signing (using equation (11)), and verification (using equation (12)). The key generation process is demonstrated in Algorithm 3.1.

Algorithm 3.1 Key generation process for the proposed method.

**Figure:**
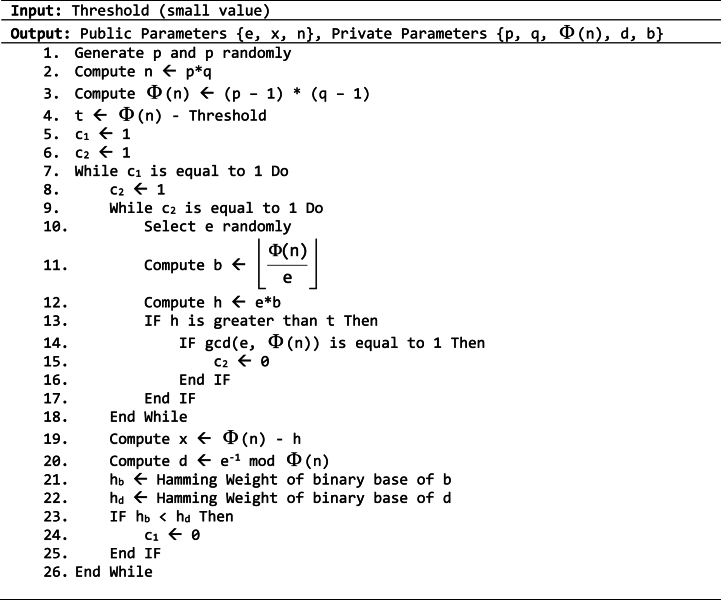

Algoritiihm 3.1 utilizes the input value as the threshold, which determines the maximum limit for *x*, ensuring it stays within the threshold. However, if the threshold value is very low, it may be harder to identify *x* that meets the conditions, especially when *n* is large. On the other hand, setting a high threshold has the benefit of simplifying the process of identifying *x* that satisfies the criterion. Nevertheless, the computation resource is increased when *x* and *b* are larger. The limitation of Algorithm 3.1 is that it depends on random search to find *b* and *x* that meet the threshold criterion. In fact, users may define *e*, *b*, and *x* by using Integer Factorization Technique to factor numbers as follows:

From Φ *(n)* *=* *eb* + *x*, assuming *x* is selected, it can be inferred that *eb* = Φ *(n)* *-* *x*. If the key creator can factorize this value, then it can be determined that:

(13) $$\Phi \left(n\right)\;–\;x=\prod_{i=1}^{l}p_{i}^{a_{i}}$$

when *p*_*i*_ is a prime number

Assuming that *x* is a small value and it is an odd positive integer, the task of factorizing Φ *(n)* - *x* is almost as difficult as factorizing *n*. On the other hand, if *x* is an even positive integer, factorization becomes easier because *2* is an assured factor of Φ *(*n*)* - *x*. Dividing Φ *(n)* *-* *x* by *2* may still result in an even positive integer, which further simplifies the factorization process. Therefore, it is preferable to choose *x* as a positive even integer.

Assuming *p*_*1*_ = *2* and Φ (*n*) – *x* = *2*^*z*^ ($\prod_{i=2}^{l}p_{i}^{a_{i}}$), then *b* can be chosen from (*2*^*z*^)*P* where *P* | $\prod_{i=2}^{l}p_{i}^{a_{i}}$. However, if *e* does not satisfy the condition *gcd(e,* Φ *(n))* *=* *1*, it can be concluded that the chosen value of *b* cannot be used, and a new value must be selected until an *e* satisfying the condition is found, as shown in Algorithm 3.2.

Algorithm 3.2 Key generation process for the proposed method (factoring technique).

**Figure:**
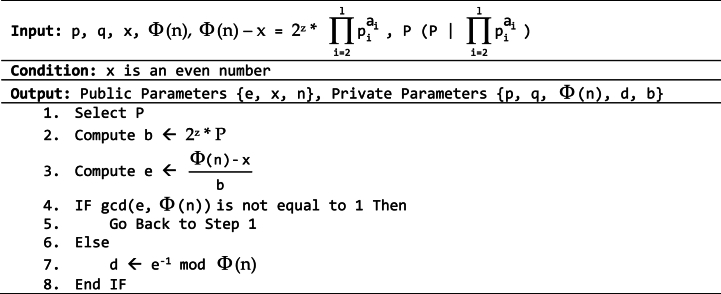

Example 2 Suppose the key generator selects *p* *=* *560753*, q *=* 830293 and *x* *=* *16.* Find *e,* *b* and *d* by using Algorithm 3.2

**Sol:** Because Φ *(n)* *=* *465587899584* and *x* = *16*, then

Φ*(*n*)* – *x* *=* *465587899568* *= (2*^*4*^*)* *(11)* *(17)* *(9283)* *(16763)*

Assume *P* *=* *(11)* *(16763)* *=* *184393* *is* *selected,* *then* *b* *=* *(2*^*4*^*)* *(184393)* *=* *2950288*

Therefore, *e* = $\frac{465587899568}{2950288}$ = *157811*

Because of *gcd(e,* Φ *(n)*) = *1*, all of the above data can be utilized, and *d* = *e*^−1^ *mod* Φ *(n)* *=* *320091496571*.

A significant limitation of utilizing Algorithm 3.2 is that, for the method to be applicable in practical situations, *n* must be assigned at least *1024* bits. However, factoring Φ *(n)* - *x* to obtain all prime factors becomes extremely difficult. In addition, in practical application, it may not be imperative to determine all prime factors. Instead, it may be suitable to calculate simply a limited number, as shown below:

From the previous example, after using some algorithms to factor Φ *(n)* - *x*, the following data may be obtained:

$$\Phi \left(n\right)-x=\left(24\right)\;\left(11\right)\;\left(17\right)\;\left(155610929\right)$$

**Note:** the example above, two prime factors of *155610929 = (9283)(16763)* are not disclosed

Therefore, *P* can be chosen as a subset of *(11) (17) (155610929)*

Assuming, *P* = *(11) (17)* *=* *187* is selected, then *b* = *(24) (187)* *=* *2992*.

Hence, *e* can be calculated as *e* = *155610929.*

Because of *gcd(e,* Φ *(n))* *=* *1*, all of the above data can be utilized, and *d* can be calculated, *d* *=* *e*^*−1*^ *mod* Φ *(n)* = *29099243537*. Moreover, it is obvious that *b* is smaller than *d*, resulting in the proposed method utilizing fewer computing resources for signing process. In fact, to prevent brute force attacks from an attacker, it is important for the key generator to avoid generating small values of *b*.

Currently, there are many techniques that have been developed for factorization, including Trial Division Algorithm (TDA), Fermat Factorization Algorithm (FFA), Pollard's *p* – *1* and Shor's algorithm. Many factorization techniques are limited to factor an integer into only two parts, which renders them ineffective to find factors of Φ *(n)* – *x*. In such cases, the result frequently comprises more than two elements. Hence, this study suggests an enhanced factorization procedure, derived from TDA, that is applicable in cases when the integer to be factored possesses several components.

Algorithm 3.3 Factorization algorithm to find result of Φ *(n)* *–* *x*.

**Figure:**
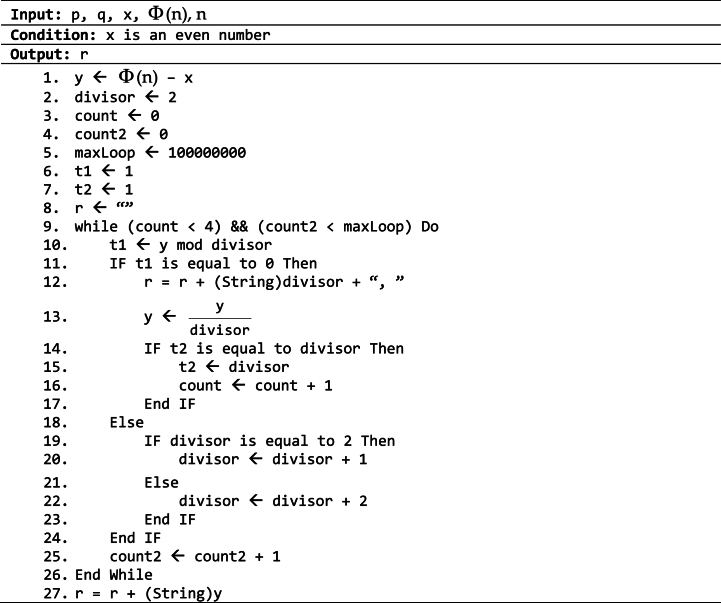

Algorithm 3.3 represents an improvement of TDA. The process is efficient when several small integers are prime factors of Φ *(n)* *–* *x*. In fact, this method does not require a detection of all prime factors. Alternatively, the method emphasizes the identification of the smallest prime factors. It evaluates a maximum of four factors. However, the integer in issue may be quite large, resulting in an extended search process. Hence, the variable maxLoop is implemented to provide the upper limit for the number of iterations utilized in the search process. If the software reaches its limit without finding an answer, it will stop running and use the most recent solution.

In addition, to reduce the amount of computational resources used in signing process, CRT can be selected instead of equation (11), as shown below:

(14) $$b_{p}=b\;\mathrm{mod}\;p\;–\;1$$

(15) $$b_{q}=b\;\mathrm{mod}\;q\;–\;1$$

Both *b*_*p*_ and *b*_*q*_ will be used as exponents to calculate *j*_*p*_ and *j*_*q*_, respectively.

(16) $$j_{p}=m^{b_{p}}\;\mathrm{mod}\;p$$

(17) $$j_{q}=m^{b_{q}}\;\mathrm{mod}\;q$$

The final step is to calculate *j* using equation (18),

(18) $$j=\left(j_{p}y_{q}q+j_{q}y_{p}p\right)\;\mathrm{mod}\;n$$

If *b* > *p* and *b* > *q*, then it is certain that *b*_*p*_ and *b*_*q*_ are smaller than *b*. However, if *b* < *p* and *b* < *q*, then *b*_*q*_ and *b*_*p*_ equal *b*. Nevertheless, it cannot be certain that applying CRT with the proposed method would be more efficient than applying CRT with RSA. Therefore, the combination between CRT and the proposed method will be chosen to finish the process when *d*_*p*_ is greater than *b*_*p*_ and *d*_*q*_ is higher than *b*_*q*_.

## 4 Experimental result

This section discusses the experiments executed with various values of *n*, including *256* bits, *512* bits, and *1024* bits. The experiments will be categorized into three segments.

The first part involves determining the average time required for the computation of all parameters associated with signing and verifying processes, using Algorithm 3.1 and Algorithm 3.2 to find all parameters. Algorithm 3.1 utilizes *threshold* = *10000* to ensure that the search for *b* and *x* is executed within the short time. In addition, *e* for each test is always *32* bits. Algorithm 3.2 considers the time required to factorize Φ *(n) - x*, selecting only even positive integers for *x* to speed up the factorization process. The factorization consists a subset of the factors of Φ *(n) - x*, including *2* and small prime factors, excluding duplicates, with a maximum of *4* factors. The second part is a comparison of the time and computational resources needed for signing process between equation (11) and equation (1). The last part describes a comparison of the time and computational resources required for verifying process between equation (12) and equation (2).

BigInteger Class is selected to develop the signing and verifying procedures. To ensure consistent parameter control, all experiments were conducted using a *2.*53 GHz Intel® Core i5 processor with 8 GB of RAM.

**Fig. 1:**
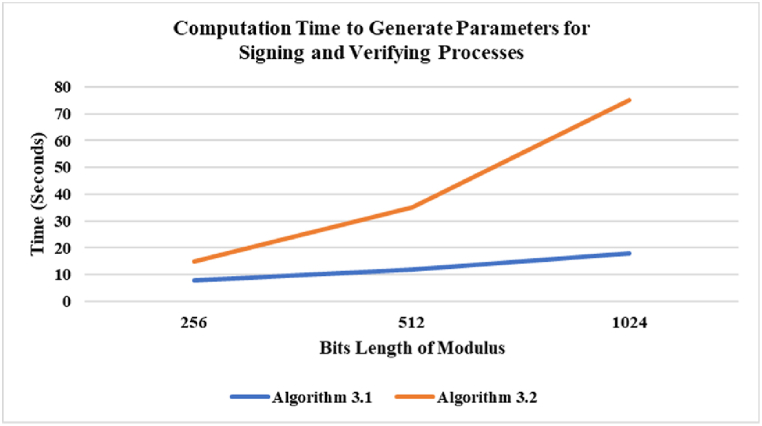
Computation time to generate parameters for signing and verifying processes.

Fig. 1 illustrates the average time expected to produce all parameters for signing and verifying processes. The experimental results indicate that Algorithm 3.1 requires less processing time than Algorithm 3.2. In fact, the benefit of using Algorithm 3.2 is the ability to choose *x*. If the proposed method uses a minimum value for *x*, it will have minimal impact on the verification component

**Fig. 2:**
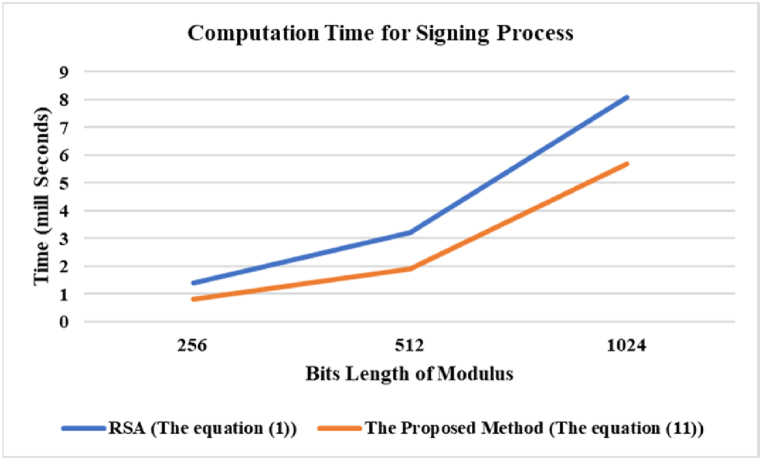
Computation time for signing process.

Fig. 2 illustrates a comparison of the average time required for digital signature generation between RSA and the suggested technique. The experimental results demonstrate that the proposed method achieves a processing speed better than equation (1), with an average time reduction of around 30 %. Although both algorithms are efficient to create digital signatures, when applied to large original files like photos [39,40] or audio [41], the data must be separated into several pieces, which necessitates executing many modular exponentiation computations. This result demonstrates that the proposed method takes less processing time than utilizing the original equation.

**Fig. 3:**
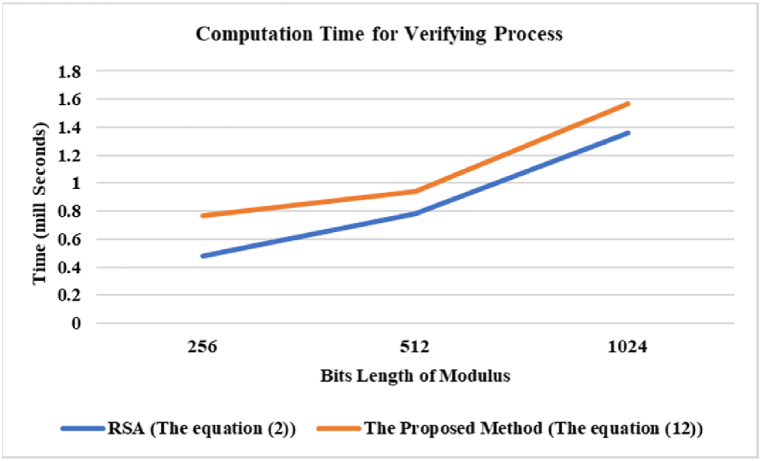
Computation time for verifying process.

Fig. 3 provides a comparison of the processing time required for verifying the digital signature between the proposed method by utilizing equation (12), and RSA by employing equation (2). The experimental results demonstrate that the proposed method requires a little increase in processing time compared to equation (2), caused by the additional computation of modular exponentiation, *m*^*x+1*^ *mod n*, which is missing in equation (2). In fact, the value of *x* is quite small (e.g., *x* < 10,000 in this experiment), leading to just a small rise in processing time.

Furthermore, the average processing time for both signing and verifying processes was examined, indicating that for *1024* bits length of *n*, the proposed method needs around *3.64* ms for each encryption-decryption cycle, in contrast to an average of *4.73* ms for RSA. This result shows that the proposed method can improve RSA. In addition, the proposed method might significantly reduce the overall processing time for multimedia data.

Because modular exponentiation is the main process for the proposed method, algorithms focused on optimizing computing resources for modular exponentiation, such as Binary Exponentiation, may be included. This improvement will reduce the processing time of the proposed method, enhancing its efficiency for practical applications.

The lower computational time of the proposed method compared to the original equation is attributed to the decreased amount of calculations for both Modular Multiplication and Modular Square operations. Example 3 is given to illustrate that the proposed method greatly decreases computational resources in comparison to the original equation.

Example 3 Given *p,* *q,* *b,* *d* and *x*, find all computational resources required for signing process and compare the performance between the original equation and the proposed method.

**Sol:**

*p* = *10098725662083958811952141541271640858154927567609846992040229699464457608923341320646038834235294442009600894421385775478077687288994524485374939088508121*

*q* = *12662705402519497004745297969423302347451021272593920920493034072440424057712736895196693230066832016077377154268524447715482415722130106707199621344645257*

*b* = *38425556356419608721452377651089154759654444892111258238632134312796233846165846102626401304457195176688769090572749860495412255343799129389573964409644988340250649070183401841348952282418609985349358955055392046710494409273893696224042306929803496794117238539709422092510798912865938604191452505331*

*d* = *107497594440122079076173784673146207228778246095911272184505010565669673190772155128074557721446311659361165222439603894107039766035216433246741246496746308125138810575569488226835351350542579847329680799474737353678841539120600572585662440908833599989695225233681881519357647061565035061759386509414676421937*

*x* = *3357* and *e* = *3327920273*

After obtaining all parameters, the necessary computational resources can be evaluated and presented in Table 1.

**Table 1:** Comparison about computation resource between RSA and the proposed method.

Algorithm	Count			
	Bit Length of the exponent	Hamming Weight of the exponent	Modular Multiplication	Modular Square
RSA	*1024*	*516*	*515*	*1023*
The Proposed Method	*992*	*486*	*485*	*991*

Table 1 presents a comparison of the computing requirements for Modular Multiplication and Modular Squaring. This table includes several parameters and illustrates Example 3, where the size of *n* is *1024* bits. In fact, it is secured for modern applications. The parameters are generated from Algorithm 3.1, providing that *b* has a size and Hamming Weight that are lower than *d*, making it appropriate for utilization. Experimental results indicate that the proposed method requires less computations for both Modular Multiplication and Modular Square in comparison to RSA.

However, considering the verification side, the additional resource is represented as *m*^*x+1*^. In this experiment, *x* *=* *3357* *=* *110100011101*_*2*_ with a size of *12* bits and Hamming Weight of *7*. Therefore, the additional computational resources are as follows: the number of computations for Modular Multiplication is *6* and the number of computations for Modular Square is *11*. Thus, considering the overall system, the proposed method saves resources as follows: the number of computations for Modular Multiplication is *24* and the number of computations for Modular Square is *21*. Therefore, considering both processes, the proposed method reduces the resources for Modular Multiplication and Modular Square by up to *45*.

## 5 Analysis

The analysis is categorized into two different cases: Case 1 examines the resources for implementing the proposed method in contrast to the original equation. Case 2 evaluates the level of security while employing the proposed method.

Case 1 In this paper, *32*-bit of *e* is chosen, adhering to the principle of selecting a reasonably small value of *e* to obtain a larger *d* for protecting against malicious attacks. From *ed* *=* *k* Φ *(n)* + *1*, *k* ≥ *1*, If *e* is already determined, it is not possible to select *k* to generate *d*. A positive correlation is observed between *k* and *d*. Conversely, the proposed method equation is *eb* = Φ *(n)* - *x*, where *k* is always *1*. Therefore, *b* is always smaller than *d*, leading to the proposed method's digital signature process demanding fewer computational resources compared to the original equation, particularly when *d* is derived from a substantial *k*, causing *d* to be considerably higher than *b*.

Case 2 Although *x* is given to be the public parameter, it is not possible for malicious entities to determine *b* without knowledge of Φ *(n)*. Therefore, the security level of the proposed technique is equivalent to RSA. However, the computation of digital signatures is significantly faster, especially for exceedingly large original communications such as photos or enormous ciphertexts.

Moreover, Wiener's attack and the modified algorithms based on continued fractions can only compute *d* but they are not applicable to find *b*. Although *d* can be computed from the relationship between *e* and *n*, this distinction renders continued fractions ineffective for directly determining *b*. To compute *b* by using continued fractions, it would be necessary to incorporate *x* in conjunction with *e* and *n*. However, no existing techniques have been identified that utilize continued fractions to derive *b* based on the relationship between these three parameters. Future works may explore the development of algorithms that leverage continued fractions for attacking the proposed method.

## 6 Conclusion

This paper presents a new approach based on RSA for generating and verifying digital signatures. The proposed method is not intended to replace RSA, but it is used selectively in replacement of RSA where analysis indicates a reduction in computational cost. Two algorithms (Algorithm 3.1 and Algorithm 3.2) designed to create all required parameters for signing and verifying processes are introduced. Both algorithms have been developed to provide parameters for the proposed method and need fewer computational resources than RSA. The reason is that the exponent utilized instead of the private key is smaller in size and has a lower Hamming weight. Although, it is time consuming by using the proposed method to generate all parameters, it is required just once. The execution of Algorithm 3.1 or Algorithm 3.2 for producing parameters provides the following experimental findings. For signing process, the proposed method utilizes fewer resources compared to the original equation. However, the proposed method requires a little more resource to verify the message, because it requires multiplying the outcome derived from the original equation. Nevertheless, the proposed algorithm overall reduces computational overhead.

## Data and code availability

Data will be made available on request. For requesting data, please write to the corresponding author.

## Declaration of competing interest

The authors declare that they have no known competing financial interests or personal relationships that could have appeared to influence the work reported in this paper.
